# The environmental toxicant 2,3,7,8-tetrachlorodibenzo-*p*-dioxin disrupts morphogenesis of the rat pre-implantation embryo

**DOI:** 10.1186/1471-213X-8-1

**Published:** 2008-01-02

**Authors:** Karla J Hutt, Zhanquan Shi, David F Albertini, Brian K Petroff

**Affiliations:** 1 The Center for Reproductive Sciences, Department of Molecular and Integrative Physiology, University of Kansas Medical Center, 3901 Rainbow Boulevard, Kansas City, KS 66160, USA; 2 Department of Internal Medicine, University of Kansas Medical Center, 3901 Rainbow Boulevard, Kansas City, KS 66160, USA; 3 Marine Biological Laboratory, Woods Hole, MA 02543, USA

## Abstract

**Background:**

Environmental toxicants, whose actions are often mediated through the aryl hydrocarbon receptor (AhR) pathway, pose risks to the health and well-being of exposed species, including humans. Of particular concern are exposures during the earliest stages of development that while failing to abrogate embryogenesis, may have long term effects on newborns or adults. The purpose of this study was to evaluate the effect of maternal exposure to the AhR-specific ligand 2,3,7,8-tetrachlorodibenzo-*p*-dioxin (TCDD) on the development of rat pre-implantation embryos with respect to nuclear and cytoskeletal architecture and cell lineage allocation.

**Results:**

We performed a systematic 3 dimensional (3D) confocal microscopy analysis of rat pre-implantation embryos following maternal exposure to environmentally relevant doses of TCDD. Both chronic (50 ng/kg/wk for 3 months) and acute (50 ng/kg and 1 μg/kg at proestrus) maternal TCDD exposure disrupted morphogenesis at the compaction stage (8–16 cell), with defects including monopolar spindle formation, f-actin capping and fragmentation due to aberrant cytokinesis. Additionally, the size, shape and position of nuclei were modified in compaction stage pre-implantation embryos collected from treated animals. Notably, maternal TCDD exposure did not compromise survival to blastocyst, which with the exception of nuclear shape, were morphologically similar to control blastocysts.

**Conclusion:**

We have identified the compaction stage of pre-implantation embryogenesis as critically sensitive to the effects of TCDD, while survival to the blastocyst stage is not compromised. To the best of our knowledge this is the first *in vivo *study to demonstrate a critical window of pre-implantation mammalian development that is vulnerable to disruption by an AhR ligand at environmentally relevant doses.

## Background

The aryl hydrocarbon receptor (AhR) pathway is a widely expressed orphan receptor pathway activated by many environmental toxicants and carcinogens. AhR ligands, including dioxins and polychlorinated biphenyls, induce a spectrum of developmental and toxic responses by modifying gene expression, altering hormonal profiles and disrupting cell proliferation and differentiation [1]. Epidemiological studies in adult human populations have linked dioxin exposure to defects in immune, neurological and reproductive function, as well as cancer [2-4]. There is now a growing concern that tissue growth and differentiation during fetal development may be especially sensitive to dioxins and dioxin-like compounds. For example, accidental exposure of human mothers to some AhR ligands has been correlated with delayed growth and development, a number of physical abnormalities, as well as intellectual and behavioral deficits in their children [5]. Moreover, animal studies show embryonic lethality, teratogenesis, cleft palate, hydronephrosis and growth retardation among the many adverse effects observed following gestational exposure to AhR ligands [6-8]. While it is accepted that maternal exposure to AhR ligands during gestation is detrimental to the health of offspring, neither the mechanism of toxicity nor the exact stages of development affected by dioxins have been fully elucidated.

Past studies examining maternal dioxin exposure and subsequent fetal health have primarily focused on post-implantation embryogenesis, while the impact of environmental contaminants on the peri-conceptional and pre-implantation period remained largely unexplored. The maternal environment during this earliest window of development has been hypothesized as critical to the long term health of offspring [9]. For example, poor maternal nutrition around the time of conception and during pre-implantation development reduces birth weight in humans [10] and animals [11] and predisposes offspring to hypertension later in life [11]. Similarly, peri-conceptional exposure of mice to environmentally relevant doses of the environmental estrogen bisphenol A induces errors in meiotic chromosome segregation, yielding embryos that survive gestation but give rise to offspring with frank genetic deficits [12]. Therefore, toxic exposure during pre-implantation embryonic development could potentially induce long term effects on fetal and offspring health.

Compacting morulae may be particularly vulnerable to the effects of environmental toxicants. Compaction is a morphogenetic process during which mammalian embryos undergo major cytoplasmic, nuclear and cytoskeletal remodeling events that lead to the establishment of apical-basal polarity [13-17]. Polarization permits the differentiative divisions that lead to the allocation of trophectoderm (TE) and the inner cell mass (ICM) for the placental and embryonic lineages, respectively [18]. Therefore, disruption of pre-implantation embryo morphogenesis by AhR ligands could conceivably have consequences for lineage allocation. This concept is supported by the finding that *in vitro *exposure of 2 cell mouse embryos to TCDD results in increased cavitation rates, a functional measure of TE differentiation [19]. Similarly, Tsutsumi et al. showed that *in vitro *exposure of 2 cell mouse pre-implantation embryos to very low levels of TCDD reduced the number of pre-implantation embryos that developed to 8 cells relative to controls, whereas blastocyst formation of the surviving 8 cell pre-implantation embryos was accelerated [20]. These studies suggest differential and stage specific effects of TCDD during pre-implantation development.

The extent to which TCDD, at environmentally relevant doses, perturbs pre-implantation mammalian development in intact reproductively fit animals has yet to be fully evaluated. Thus, in a well-established rat model, we studied the effect of maternal TCDD exposure on early embryogenesis with respect to blastomere nuclear and cyto-architecture. We show specific nuclear and cytoskeletal modifications revealed from a systematic 3D confocal microscopy analysis of rat pre-implantation embryos following maternal exposure to TCDD. Both chronic and acute maternal TCDD exposure disrupted morphogenesis at the compaction stage (8–16 cell), with defects including monopolar spindle formation, f-actin capping, aberrant cytokinesis and distortion of nuclear shape and position. Notably, maternal TCDD exposure did not compromise survival to blastocyst, which with the exception of nuclear shape, were morphologically similar to control blastocysts. These studies raise further concerns regarding the consequences of early embryo exposures to prevalent environmental toxicants like TCDD.

## Results

### Chronic maternal TCDD exposure disrupts 8–16 cell pre-implantation embryo morphogenesis

**Figure 1:**
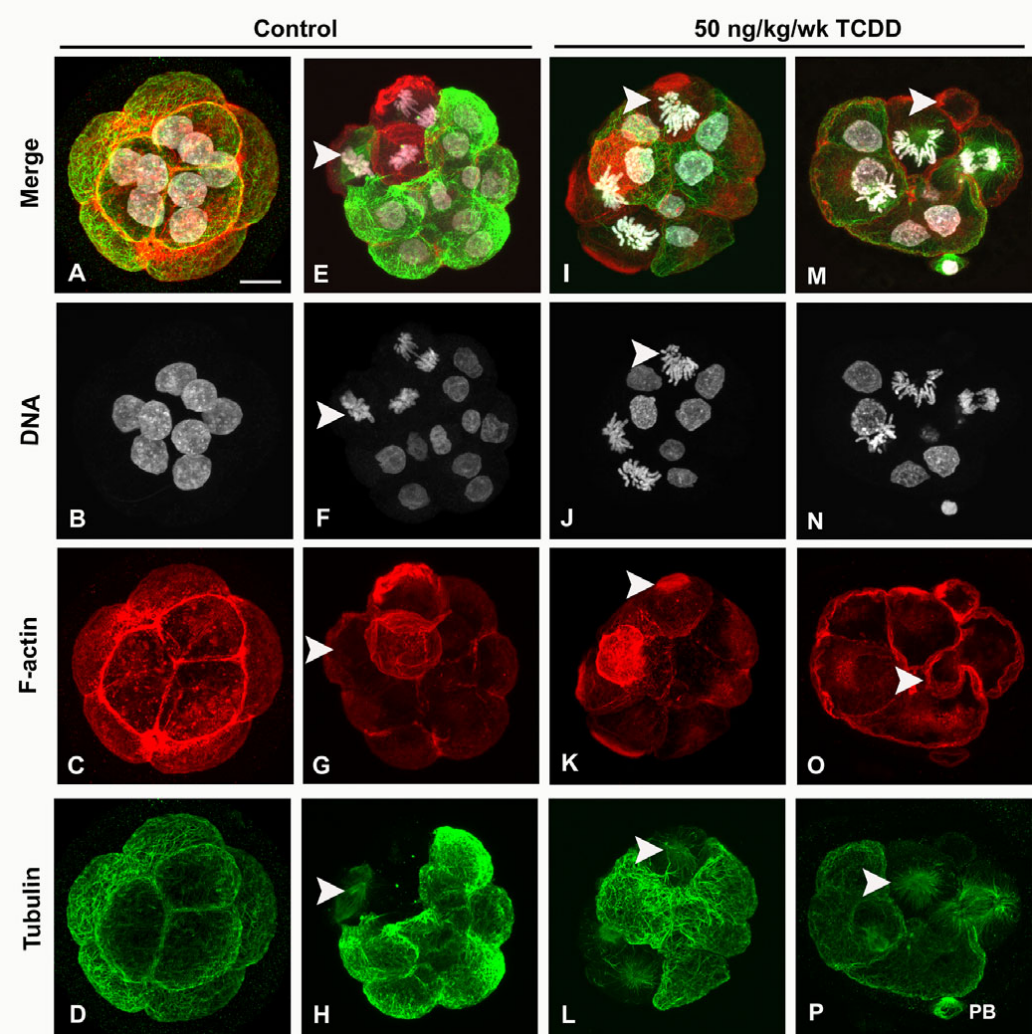
**Chronic maternal TCDD exposure induces nuclear and cytoskeletal defects in compaction stage pre-implantation embryos**. Compaction stage pre-implantation embryos from control and chronically exposed (50 ng/kg/wk TCDD) female rats were processed for visualization of microtubules, f-actin and DNA by confocal microscopy. (A-D) Control 8-cell pre-implantation embryo with blastomeres of similar size and shape, basally positioned interphase nuclei and cytoplasmic microtubule arrays. F-actin is distributed at the cell cortex. (E-H) Control 12-cell pre-implantation embryo with a normal bipolar mitotic spindle (E and H, arrows), metaphase chromosome configuration (F, arrow) and cortical f-actin localization (G, arrow). (I-L) 50 ng/kg/wk TCDD exposed 9-cell pre-implantation embryo with abnormal mitotic spindles (L, arrow) and metaphase chromosome configurations (J, arrow), and enhanced f-actin cortical localization (K, arrow) in multiple blastomeres. (M-P) 50 ng/kg/wk TCDD exposed 8-cell pre-implantation embryo with an anucleate fragment (M, arrow) and abnormal cytokinesis (O, arrows). Monopolar spindle (P, arrow). PB, polar body. Scale bar: 15 μm.

We first asked whether chronic maternal exposure to TCDD affected the number of pre-implantation embryos relative to controls. As shown in Table 1, exposure to TCDD had no effect on pre-implantation embryo number, suggesting that ovulation, fertilization efficiency and embryonic survival were not influenced at these doses. These findings are consistent with previous studies demonstrating embryotoxicity only at considerably higher doses of TCDD [21]. Approximately 80% of pre-implantation embryos from control rats were morphologically normal 8–16 cell stage concepti, with regular shaped and sized blastomeres and no nuclear or cellular fragmentation (Table 1, Fig. 1A–H, Additional file 1). The apical and basolateral surfaces of each blastomere were smooth and the overall structure of controls was highly organized with respect to the relative position of blastomeres. Interphase blastomeres from controls had cytoplasmic microtubule networks, basally positioned nuclei and apically polarized f-actin localization. Centrally positioned bipolar spindles were consistently observed in mitotic blastomeres of control pre-implantation embryos (Fig. 1E–H, arrow).

**Table 1:** Numbers and health status of pre-implantation embryos collected following chronic and acute maternal TCDD exposure

Treatment (# animals)	Average # embryos/animal (range)	Average # blastomere/embryo (range)	# Normal embryos/total embryos (%)
Chronic exposure (compaction)			
Control (3)	13.0 (12–14)	12.5 (7–18)	31/39 (79.5)
50 ng/kg/wk TCDD (3)	13.7 (13–15)	11.3 (4–17)	15/41 (36.6)*
Acute exposure (compaction)			
Control (3)	13.3 (13–14)	9.5 (3–16)	32/40 (80.0)
50 ng/kg TCDD (5)	12.0 (11–13)	9.5 (3–16)	31/60 (51.7)*
1 μg/kg TCDD (6)	9.2 (3–13)	10.1 (6–16)	21/46 (45.7)*
Acute exposure (blastocyst)			
Control (6)	10.5 (9–12)	31.9 (13–52)	48/63 (76.2)
50 ng/kg TCDD (3)	9.7 (9–10)	32.1 (8–58)	17/29 (58.6)
1 μg/kg TCDD (5)	9.0 (9–12)	37.5 (8–63)	20/45 (45.5)*

*Significantly different from control

**Figure 2:**
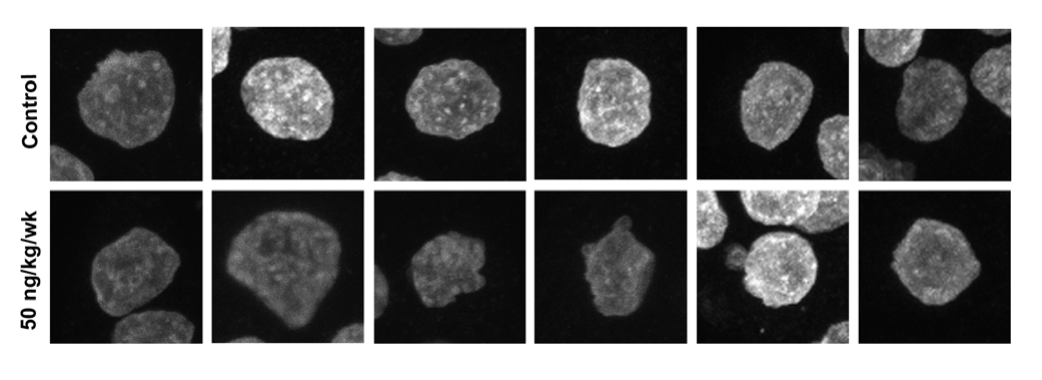
**Nuclear profiles of compaction stage pre-implantation embryos from control and chronically exposed animals**. Z-series datasets for the DNA channel were compressed into a single plane and 6 randomly selected nuclei (each from a different pre-implantation embryo) for each group (Control and 50 ng/kg/wk TCDD) were compared.

In contrast, significantly lower proportions (~37%) of pre-implantation embryos from chronically exposed dams were morphologically normal (Table 1). Embryos from chronically exposed dams exhibited a range of defects, including irregularly sized and shaped blastomeres (Fig. 1I–P, Additional file 2). Moreover, f-actin staining at the apical and basolateral boundary of compacted blastomeres was highly irregular (Fig. 1O). Analysis of interphase blastomere nuclei also revealed alterations in shape not evident in controls (Fig. 2; Additional files 3 and 4). In many cases these nuclei had an irregular boundary with one or more prominent projections from the nuclear surface. In addition to a more central nuclear position, chronic TCDD exposure impeded chromosome segregation or cytokinesis resulting in binucleate cells, anucleate fragments and cells containing large nuclei or micronuclei (Fig. 1M–P; Additional file 2). In fact, pre-implantation embryos exposed to TCDD frequently exhibited monopolar spindles (13/18 spindles were monopolar). The solitary spindle pole was usually oriented toward the apical surface of blastomeres, with poorly aligned chromosomes located at the distal ends of microtubules (Fig. 1I–P, Additional file 2). In mitotic blastomeres, aberrant f-actin caps were adjacent to the spindle pole (Fig. 1I–P).

### Acute periconceptional TCDD exposure disrupts 8–16 cell pre-implantation embryo morphogenesis

These initial findings prompted further analysis of cytoskeletal and nuclear characteristics in pre-implantation embryos to determine if limiting TCDD exposure to the time between oocyte maturation, ovulation and implantation (i.e. the periconceptional period) would similarly modify pre-implantation embryo organization. Mature naturally cycling female rats were exposed to a single dose of TCDD (50 ng/kg or 1 μg/kg) or vehicle on the evening of proestrus and compaction (8–16 cell) and early blastocyst stage (32 cell or more) pre-implantation embryos were collected and analyzed.

**Figure 3:**
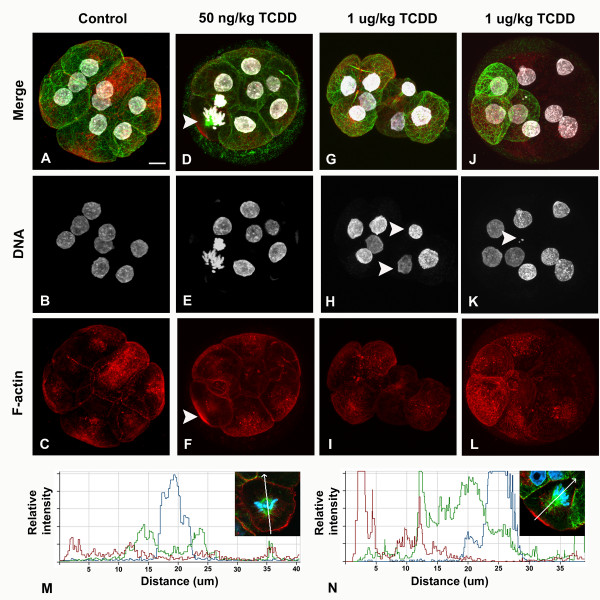
**Acute maternal periconceptional TCDD exposure induces nuclear and cytoskeletal defects in compaction stage pre-implantation embryos**. Compaction stage pre-implantation embryos collected from control and acutely exposed (50 ng/kg and 1 μg/kg TCDD) female rats and processed for visualization of microtubules, f-actin and DNA by confocal microscopy. (A-C) Control 8-cell pre-implantation embryo. (D-F) 50 ng/kg TCDD exposed 8-cell pre-implantation embryo with monopolar spindle (D, arrow) and f-actin cortical localization (F, arrow). (G-I) 1 μg/kg TCDD exposed 8-cell pre-implantation embryo with distorted overall shape and centrally localized nuclei (H, arrows). (J-L) 1 μg/kg TCDD exposed 8-cell pre-implantation embryo with global defects in the microtubule and f-actin networks; note micronuclei (K, arrow). Relative fluorescent intensity profiles for a normal bipolar spindle from a control pre-implantation embryo (M) and a monopolar spindle from a 50 ng/kg TCDD exposed pre-implantation embryo (N) are shown. Note the absence of tubulin (green) fluorescence on the basal side of the aligned chromosomes (blue) and the intense f-actin (red) signal at the apical cortex compared to the basal cortex (N). This is a different embryo to that shown in D-F. Scale bar: 15 μm.

**Figure 4:**
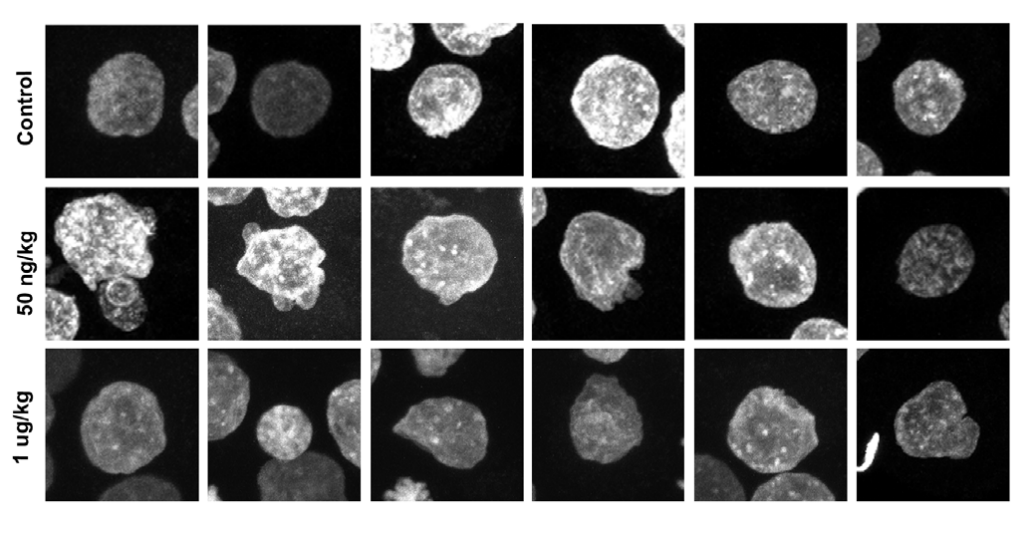
**Nuclear profiles of compaction stage pre-implantation embryos from control and acutely exposed animals**. Z-series datasets for the DNA channel were compressed into a single plane and 6 randomly selected nuclei (each from a different pre-implantation embryo) for each group (Control and 50 ng/kg and 1 μg/kg TCDD) were compared.

Acute maternal TCDD exposure at the lower dose did not alter the number of compaction stage pre-implantation embryos relative to controls, but only ~52% of pre-implantation embryos from treated animals were normal (Table 1). Exposure to the higher dose of TCDD decreased the number of pre-implantation embryos, and an even lower proportion (~46%) of these pre-implantation embryos were normal (Table 1). A range of defects in nuclear and cytoskeletal integrity were observed (Fig. 3D–L), including a dose dependent loss of microtubule and f-actin staining in some blastomeres (Fig. 3J–L). Additionally, f-actin localization changed from a plasma membrane concentrated (Fig. 3C) to a more diffuse pattern of stain (Fig. 3I and 3L). In pre-implantation embryos from the 1 μg/kg treatment group, blastomeres often exhibited micronuclei (Fig. 3K, arrow) and nuclei of different sizes. Further analyses of nuclear shape revealed a range of profiles deviating from being smooth surfaced in controls to more irregular contours detected in blastomere nuclei from either treatment group (Fig. 4). Again, monopolar spindles with intense f-actin caps were evident at both low and high doses of TCDD (12/17 and 7/11 spindles, respectively, were monopolar) (Fig. 3D–F). This was further confirmed by using pixel intensity line scans to monitor the topography of cortical f-actin staining in mitotic cells (Fig. 3M and 3N). These analyses revealed equivalent intensity of f-actin around central spindles in control cells. However, as much as a five-fold increase at the apical f-actin caps was detected in cells with monopolar spindles relative to the opposite side of the blastomere (Fig. 3M and 3N). Frequently, astral-like microtubule fibers were detected between the spindle pole and cortex, accentuating the asymmetric displacement of chromosomes seen in pre-implantation embryos from treated animals (Fig. 3N).

### Acute periconceptional TCDD exposure permits pre-implantation embryo survival to blastocyst

We then asked whether the striking modifications evident at compaction were propagated through to the early blastocyst stage. In particular, it seemed likely that the occurrence of monopolar spindles would abrogate efficient cell cycle progression and negatively impact further pre-implantation embryo development. However, exposure to TCDD affected neither the number of pre-implantation embryos surviving to blastocyst nor the average number of cells within each blastocyst (Table 1), suggesting a normal rate of cell cycle progression in treated pre-implantation embryos.

**Figure 5:**
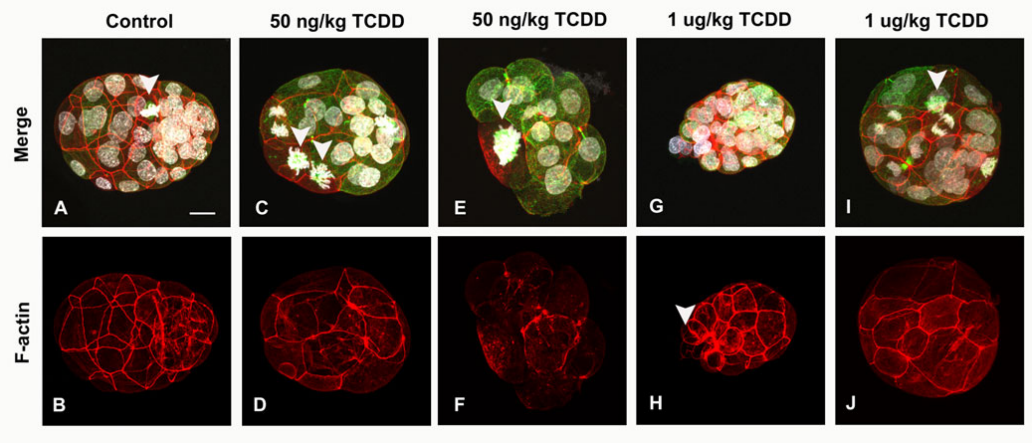
**Acute maternal periconceptional TCDD exposure induces nuclear and cytoskeletal defects in early blastocysts**. Blastocysts collected from control and acutely exposed (50 ng/kg or 1 μg/kg TCDD) female rats were processed for visualization of microtubules, f-actin and DNA by fluorescence confocal microscopy. (A, B) Control blastocyst with blastocoel and distinct ICM and TE cell populations; note normal bipolar spindle (arrow). (C, D) 50 ng/kg TCDD exposed blastocyst with an abnormal metaphase blastomere (arrow) and slightly distorted cell shapes. (E, F) Developmentally delayed 50 ng/kg TCDD exposed blastocyst with an abnormally large mitotic spindle (arrow). (G, H) 1 μg/kg TCDD exposed blastocyst with a compacted morphology. F-actin and tubulin disorganization is apparent at one pole (arrow). (I, J) 1 μg/kg TCDD exposed blastocyst with multipolar spindle (arrow) and irregular sized cells. Scale bar: 15 μm.

TCDD exposure at the higher dose did decrease the number of blastocysts exhibiting normal morphology (~46%) (Table 1), though TCDD associated abnormalities at the blastocyst stage were considerably less severe than those observed at the 8–16 cell stage. Blastocysts obtained from control animals typically contained 32 cells or more, formed blastocoel cavities and were comprised of cells with the distinct morphology of both TE and ICM (Fig. 5A and 5B; Additional file 5). The majority of blastocysts from treated animals also attained this general morphology (Additional files 6 and 7). However, approximately half of the blastocysts exhibited discernable defects in one or more outer cells following TCDD exposure. Amongst these defects, cells failed to assume the squamous morphology typical of TE, exhibited a loss of interphase microtubule and f-actin networks and were binucleate or contained multipolar spindles (Fig. 5C–J). However, such cytoskeletal and nuclear defects were usually observed within individual blastomeres of otherwise normal appearing blastocysts. Interestingly monopolar spindles were only rarely observed in blastocysts from treated animals (Fig. 5C). Control and treated blastomeres exhibited a low level of pyknotic nuclei, which was restricted to the ICM. The number of blastomeres with pyknotic nuclei was similar in control and treated blastocysts.

**Figure 6:**
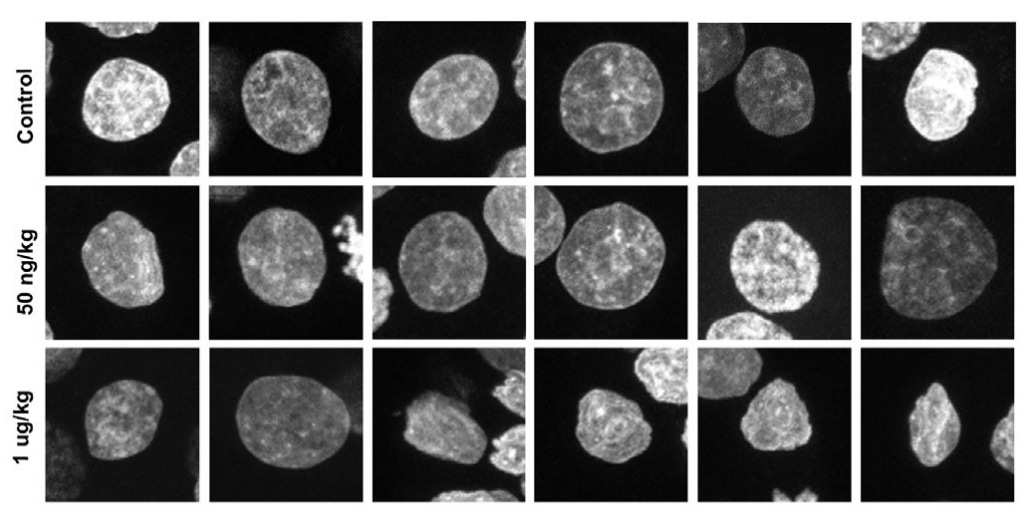
**Blastocyst nuclear profiles from control and acutely exposed animals**. Z-series datasets for the DNA channel were compressed into a single plane and 6 randomly selected nuclei (each from a different blastocyst) for each group (Control, 50 ng/wk and 1 μg/kg TCDD) were compared. Only mural TE were analyzed.

In comparing nuclear structure among blastocysts, it was noted that while TE cells contained nuclei resembling controls in the 50 ng/kg TCDD exposed group, at higher concentrations (1 μg/kg TCDD) nuclei were conspicuously smaller and irregular in shape (Fig. 6). Despite this effect, TE cells from all groups exhibited prominent f-actin boundaries and given the development of the blastocoel, we concluded that this subpopulation of cells had differentiated into highly polarized epithelium able to support fluid transport. The consequences of maternal TCDD exposure on the ICM nuclear structure remains to be established.

## Discussion

Exposure to environmental toxicants, such as TCDD, prior to and during the earliest stages of pregnancy has been linked to developmental disabilities after birth in both human and animal studies [5-8]. We conducted a systematic high resolution confocal microscopy analysis of rat pre-implantation embryos that has revealed previously unappreciated morphogenetic defects following maternal TCDD exposure. We have shown that both chronic and acute maternal exposure to TCDD induced nuclear and cytoskeletal defects in pre-implantation embryo morphogenesis. Specifically, our studies revealed effects of TCDD on 1) mitotic spindle integrity, 2) chromosome alignment, 3) nuclear and cellular size and shape and 4) cytokinesis efficiency. Furthermore, we have identified the compaction stage of pre-implantation embryogenesis as critically sensitive to the effects of TCDD, while survival and development to the blastocyst stage is not compromised. To the best of our knowledge, this is the first *in vivo *study to demonstrate a critical window of early mammalian development that is vulnerable to disruption by an AhR ligand at environmentally relevant doses.

The comparison of acute verses chronic maternal exposure to TCDD on subsequent embryo quality in the current study encompasses examination of both dose effects and transgenerational toxicant actions. Dams in the acute exposure paradigm were exposed to low and high doses of TCDD limited to a single administration immediately preceding ovulation. The half life of TCDD in the rat is approximately 3 weeks, implying significant exposure until collection of the embryo. In the chronic exposure model, continuous exposure to TCDD in the dam began *in utero *and continued until breeding sacrifice at 3 months of age. In other words, the acute exposures were comprised of exposures to both the mother and the embryo, while the chronic exposure entails exposure of grandmother, mother and offspring. The results presented here show that both acute and chronic TCDD treatment protocols significantly compromised embryo quality.

It is likely that pre-implantation embryos are a direct target for TCDD, given that AhR is expressed throughout pre-impanation development [22] and that *in vitro *exposure of mouse pre-implantation embryos to TCDD accelerates differentiation of the blastocyst [19,20]. However, due to the *in vivo *experimental design employed in this study, we can not rule out effects of TCDD on the oocyte nor on the mother's physiology, as factors contributing to the outcomes realized during pre-implantation embryo development. AhR ligands are known endocrine disruptors [23] and have also been shown to compromise oocyte quality by inducing apoptosis in cumulus cells [24]. Additionally, recent studies have suggested that TCDD may actually accumulate in the follicular fluid and in the uterus, emphasizing the importance of the mother's physiology in contributing to pre-implantation embryo health and the necessity for *in vivo *studies [20,25].

One of the most striking effects of TCDD, at both doses, was the induction of aberrant mitotic spindles and a failure in chromosome alignment in compaction stage pre-implantation embryos. AhR ligands, such as TCDD, may be involved in generating meiotic spindle aberrations by causing local increases in the concentration of 2-methoxyestradiol (2-ME) [26]. 2-ME binds tubulin and influences microtubule polymerization and function. It is an endogenous metabolite of 17β-estradiol normally present within granulosa cells and follicular fluid of the ovary. Exposure to elevated levels of 2-ME causes spindle abnormalities, chromosome congression failure and nondisjunction in mouse oocytes and it is conceivable that such abnormalities would be further propagated within the developing embryo. Interestingly, exposure of bovine pre-implantation embryos to 2-ME does not inhibit passage from morula to blastocyst, and the cell cycle proceeds despite aberrations in spindle morphology [27].

Similarly, exposure to TCDD did not inhibit development to blastocyst and the data presented here clearly demonstrate that the overall structure and morphology of treated blastocysts were similar to control blastocysts. However, the widespread prevalence of defects uncovered in compaction stage pre-implantation embryos of treated animals, together with the relative paucity of cells exhibiting defects at the blastocyst stage, is disconcerting. Thus, a central question raised by this work is "What is the fate of aberrant compaction stage blastomeres?" We suggest three scenarios that may contribute to the survivability of TCDD exposed pre-implantation embryos. Firstly, defective cells may be eliminated by apoptosis during the transition from compacted pre-implantation embryo to blastocyst. Brison and Shultz showed apoptosis only occurs after compaction and is predominantly located in the ICM of the mouse [28]. However, TCDD exposure did not increase the incidence of pyknosis in pre-implantation embryos in this study and thus elimination of TCDD-induced defects by selective apoptosis seems unlikely. This is consistent with an earlier study in which *in vitro *exposure of 2, 4, or 8 cell mouse pre-implantation embryos to TCDD did not significantly increase the number of TUNEL positive cells, alter the Bax/Bcl-2 expression ratio, or change cell number at the blastocyst stage [22].

Alternatively, defective cells may initiate repair mechanisms to rectify errors. Surveillance mechanisms that alert cells to impending errors in chromosome segregation exist in many normal somatic cells exhibiting a stable euploid condition. Such cell cycle checkpoints are engaged in response to structural aberrations as wide-ranging as chromosome misalignment to centrosome number [29]. Notably, monopolar spindles with poorly aligned chromosomes are defects likely to activate cell cycle checkpoints. However, knockout and transgenic studies suggest that checkpoint controls may not be operational until the time of implantation in mouse embryos [30]. Furthermore, lack of checkpoint activity in human embryos is suggested by the extreme degree of aneuploidy and mixoploidy seen in association with defects in mitotic spindle organization [31,32]. If cell cycle checkpoints were activated in rat pre-implantation embryos, one might expect either an increase in the mitotic index or decrease in total cell number to be evident in blastocysts derived from TCDD exposed animals compared to controls. While there was no obvious increase in the presence of mitotic figures or cell number, further studies will be required to fully investigate this possibility.

It is possible that our detection of monopolar spindles was biased by a prolonged prometaphase delay that yielded euploid daughter cells after a correction that we were unable to detect in fixed samples. While this seems unlikely given the similar distribution of M-phase stages observed in control and treated pre-implantation embryos, on-going live cell recording experiments should resolve this dilemma. Moreover, cytogenetic assays will be needed to establish the incidence of aneuploidy, known to be elevated in human embryos exhibiting similar spindle defects, to better understand the status of checkpoint controls during this critical juncture during mammalian embryogenesis.

A final possibility is retention of defective blastomeres and their contribution to either or both of the lineages established in the blastocyst. While it is interesting to speculate that the TE lineage naturally undergoes ploidy variations indicative of less stringent cell cycle checkpoint surveillance [33], our data to date cannot rigorously assign fates to the aberrant blastomeres present during compaction. Given the changes in nuclear shape and the cytoskeleton resulting from exposure to TCDD during oocyte maturation and pre-implantation development, the emergence of cell polarity and lineage assignment during the compaction process may be impacted in subtle ways and yet still have major consequences for the later stages of embryogenesis. For example, the disruption of nuclear architecture is the central factor underlying a physiologically diverse group of inherited diseases known as laminopathies [34]. Moreover, the epigenetic assignment of blastomeres to TE or ICM immediately precede the time we report here to be most sensitive to the effects of TCDD [35]. In this regard, Skinner and colleagues have recently demonstrated altered epigenetic marks following exposure to environmental estrogens leading to disease phenotypes in adult male offspring that were passed on to subsequent generations [36]. It was also recently shown that *in vitro *exposure of mouse pre-implantation embryos to TCDD increased methyl transferase activity, altered the methylation status of imprinted genes *H19 *and *Igf2 *and retarded subsequent fetal growth [37]. Thus, disruption of epigenetic programming made during compaction provides a plausible mechanism by which pre-implantation exposure to TCDD could affect later development. In this light, the persistence of nuclear defects in shape and position may be subtle indicators of disruption of chromatin remodeling and epigenetic reprogramming during oocyte maturation and development up to compaction.

## Conclusion

We have observed that compaction stage pre-implantation embryogenesis as critically sensitive to the effects of TCDD, while survival to the blastocyst stage is not compromised. The present work assumes particular relevance when considered together with recent evidence suggesting long term impacts on the health and well-being of offspring following environmental perturbations during the periconceptional and pre-implantation period [11,38]. While the pre-implantation embryo may exhibit resiliency and plasticity in obtaining a state of implantation competence, perturbations during this critical window of development may be propagated with further cellular expansion and could abrogate developmental processes that will only surface after birth. Identifying the specific targets of TCDD in the pre-implantation embryo, especially those that link cell cycle control with cytoskeletal and nuclear remodeling, represents an important avenue for future investigation.

## Methods

### Animals

Female Sprague-Dawley rats (Charles River Laboratories) were housed under a 12L:12D photoperiod at an ambient temperature of 23 ± 2°C, with food and water *ad libitum*. All procedures were approved by the University of Kansas Medical Center Institutional Animal Care and Use Committee. In all experiments, pre-implantation embryos were obtained from naturally mated rats. Estrus cycles were monitored by vaginal cytology, with normal estrous cycle duration of 4–5 days [39]. Source and purity of TCDD: CAS 1746-01-6; MW, 321.9; purity, > 99%.

### Experimental design

In experiment 1, rats were exposed chronically to doses of TCDD that mimic exposure of high risk populations in humans [40,41]. Weekly oral dosing was used in a previously validated regime [21,42]. Initially, pregnant dams (n = 3 for each experimental group) received an oral dosing of TCDD (50 ng/kg) or corn oil vehicle (4 ml/kg) on day 14 and 21 of gestation and then on days 7 and 14 postpartum to provide in utero and postnatal lactational exposure, respectively. On postnatal day 21, female pups (n = 3 per experimental group) were weaned and orally dosed with TCDD (50 ng/kg) or corn oil vehicle (4 ml/kg), with dosing continued at weekly intervals thereafter. At 3 months of age, proven males were introduced on the evening of proestrus and mating was confirmed by the presence of sperm on vaginal cytology the following morning. Pre-implantation embryos were collected in FHM (Chemicon) media pre-warmed to 37°C by flushing oviducts and uteri on day 4.5 post coitum.

In experiment 2, female Sprague-Dawley rats (n = 3–6 for each experimental group) received a single oral dose (50 ng/kg or 1 μg/kg) of TCDD or corn oil vehicle (4 ml/kg) on the evening of proestrus and were housed with males of proven fertility. At the time of dosing rats were 50 days of age. Again, mating was confirmed by the presence of sperm on vaginal cytology the following morning. Pre-implantation embryos were collected in FHM (Chemicon) media pre-warmed to 37°C by flushing oviducts and uteri on day 4.5 or 5.5 post mating.

### Immunofluorescence

Pre-implantation embryos were processed for microtubule, DNA and f-actin immunofluorescence as previously described [43]. Immediately following their collection, pre-implantation embryos were fixed for 30 min in 4% PFA at 37°C and stored at 4°C in wash solution comprising PBS supplemented with 2% BSA, 2% skim milk powder, 2% normal goat serum, 100 mM glycine, 0.01% Triton-X-100 and 0.2% sodium azide until processing for immunofluorescence. Pre-implantation embryos were extracted for 30 min at room temperature in 0.1% Triton-X-100 and incubated overnight at 4°C in wash solution. For immunostaining of microtubules, embryos were first incubated with mouse monoclonal anti-αβ tubulin (Sigma) diluted 1:100 in wash solution for 1 h at 37°C, followed by Alexa 488 labeled goat anti-mouse IgG (Molecular Probes) diluted 1:1000 in wash solution for 1 h at 37°C. DNA was stained with Hoechst 33258 (1 μg/ml in wash solution) for 30 min and f-actin integrity was analyzed by staining with rhodamine labeled phalloidin (1 μg/ml in wash solution; Molecular Probes) for 30 min. Pre-implantation embryos were mounted under cover slips without compression in medium containing 50% glycerol and 25 mg/ml sodium azide.

Pre-implantation embryos were analyzed on a Zeiss LSM Pascal confocal imaging system mounted on a Zeiss Axioscope II using UV (405 nm), HeNe (543 nm) and Argon (488 nm) laser excitation. For every embryo, a complete Z-axis data set was collected at 0.8 μm intervals (~50 sections/embryo) using a x63 oil objective (na = 1.4). Laser power, gain and offset settings were not changed during acquisition. Line scans and spatial restoration and 3 dimensional projections for each Z-series data set were computed and analyzed using Zeiss LSM 5 Image Browser.

### Classification of pre-implantation embryos

Pre-implantation embryos were classified as abnormal if they contained blastomeres exhibiting one or more of the following: irregular size, irregular shape, weak or undetectable f-actin or tubulin, cellular fragmentation or micronuclei. Additionally, blastomeres containing metaphase-like chromosomes with mitotic spindles absent, or deviating from a focused bipolar microtubule array, were considered abnormal.

### Statistical analysis

Chi-square was used to analyze the proportion of normal and abnormal pre-implantation embryos. P-values of less than 0.05 were considered significant.

## Authors' contributions

KJH carried out the embryo collection, analysis and interpretation of the data and drafted the manuscript. ZS dosed the animals and collected the embryos. DFA analyzed and interpreted the data and helped to draft the manuscript. BKP conceived of the study and participated in its design and coordination and helped to draft the manuscript. All authors read and approved the final manuscript.

## Supplementary Material

Additional file 1 **Control compaction stage pre-implantation embryo**. Z-axis step through of control pre-implantation embryo. Green: tubulin; red: f-actin; white: DNA. Click here for file

Additional file 2 **Chronically treated compaction stage pre-implantation embryo**. Z-axis step through of a pre-implantation embryo following chronic maternal exposure to TCDD (50 ng/kg/wk). This is the same pre-implantation embryo as shown in Fig. 1M–P. Green: tubulin; red: f-actin; white: DNA. Click here for file

Additional file 3 **Nuclear profile of compaction stage pre-implantation embryos from control animal**. 3D rotation illustrating the nuclear profile of a control pre-implantation embryo. Click here for file

Additional file 4 **Nuclear profile of compaction stage pre-implantation embryos from chronically exposed animal**. 3D rotation illustrating the nuclear profile of a pre-implantation embryo following chronic maternal exposure to TCDD (50 ng/kg/wk). Click here for file

Additional file 5 **Control blastocyst**. 3D rotation illustrating the nuclear and f-actin profiles of a control blastocyst. This is the same embryo as shown in Fig. 3A and 3B. Red: f-actin; white: DNA. Click here for file

Additional file 6 **Acute 50 ng/kg blastocyst**. 3D rotation illustrating the nuclear and f-actin profiles of a blastocyst following acute exposure to 50 ng/kg TCDD. Red: f-actin; white: DNA. Click here for file

Additional file 7 **Acute 1 μg/kg blastocyst**. 3D rotation illustrating the nuclear and f-actin profiles of a blastocyst following acute exposure to 1 μg/kg TCDD. Red: f-actin; white: DNA. Click here for file
